# Development and External Validation of a Machine Learning Model for Progression of CKD

**DOI:** 10.1016/j.ekir.2022.05.004

**Published:** 2022-05-13

**Authors:** Thomas Ferguson, Pietro Ravani, Manish M. Sood, Alix Clarke, Paul Komenda, Claudio Rigatto, Navdeep Tangri

**Affiliations:** 1 Department of Internal Medicine, Max Rady College of Medicine, University of Manitoba, Winnipeg, Manitoba, Canada; 2 Seven Oaks Hospital Chronic Disease Innovation Centre, Winnipeg, Manitoba, Canada; 3 Department of Medicine, Cumming School of Medicine, University of Calgary, Calgary, Alberta, Canada; 4 Department of Community Health Science, Cumming School of Medicine, University of Calgary, Calgary, Alberta, Canada; 5 Department of Medicine and the Ottawa Hospital Research Institute, The Ottawa Hospital, Ottawa, Canada

**Keywords:** CKD progression, machine learning, predictive modeling

## Abstract

**Introduction:**

Prediction of disease progression at all stages of chronic kidney disease (CKD) may help improve patient outcomes. As such, we aimed to develop and externally validate a random forest model to predict progression of CKD using demographics and laboratory data.

**Methods:**

The model was developed in a population-based cohort from Manitoba, Canada, between April 1, 2006, and December 31, 2016, with external validation in Alberta, Canada. A total of 77,196 individuals with an estimated glomerular filtration rate (eGFR) > 10 ml/min per 1.73 m^2^ and a urine albumin-to-creatinine ratio (ACR) available were included from Manitoba and 107,097 from Alberta. We considered >80 laboratory features, including analytes from complete blood cell counts, chemistry panels, liver enzymes, urine analysis, and quantification of urine albumin and protein. The primary outcome in our study was a 40% decline in eGFR or kidney failure. We assessed model discrimination using the area under the receiver operating characteristic curve (AUC) and calibration using plots of observed and predicted risks.

**Results:**

The final model achieved an AUC of 0.88 (95% CI 0.87–0.89) at 2 years and 0.84 (0.83–0.85) at 5 years in internal testing. Discrimination and calibration were preserved in the external validation data set with AUC scores of 0.87 (0.86–0.88) at 2 years and 0.84 (0.84–0.86) at 5 years. The top 30% of individuals predicted as high risk and intermediate risk represent 87% of CKD progression events in 2 years and 77% of progression events in 5 years.

**Conclusion:**

A machine learning model that leverages routinely collected laboratory data can predict eGFR decline or kidney failure with accuracy.

## Graphical abstract

**Figure:**
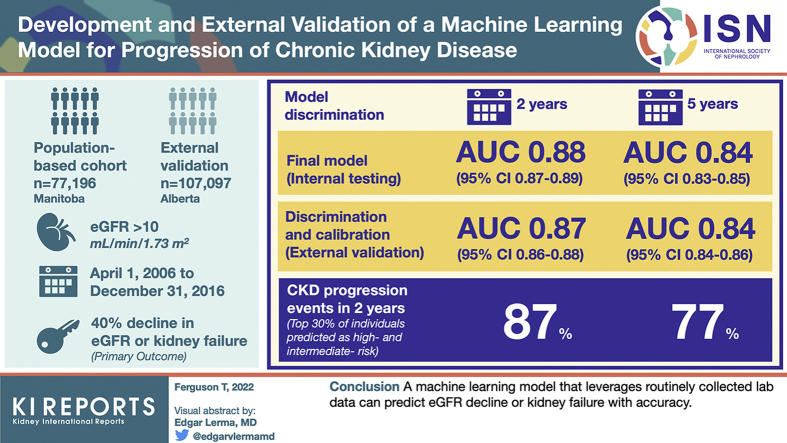

CKD currently affects >850 million adults worldwide and is associated with increased morbidity and mortality and high health care costs.^1^ Although only a few patients with CKD will develop kidney failure, much of the excess morbidity and costs associated with CKD are driven by individuals who progress to more advanced stages of CKD before reaching organ failure requiring dialysis.^2^ Accurate prediction of individual risk of CKD progression could improve patient experiences and outcomes through knowledge sharing and shared decision-making with patients,^3^ enhance care by better matching the risks and harms of therapy to the risk of disease progression,^4^ and through improved health system efficiency resulting from better alignment between resource allocation and individual risk.

The Kidney Failure Risk Equation is an internationally validated risk predictieon equation that accurately predicts the risk of progression to kidney failure for an individual patient with CKD. However, this equation has important limitations in that it applies only to later stages of CKD (G3–G5) and considers only the outcome of kidney failure requiring dialysis. In earlier stages of CKD, kidney failure is a rare event, even if progression to a more advanced stage is not. In these early stages, a decline in GFR of 40% is both clinically meaningful to patients and physicians and allows sponsors to design feasible randomized controlled trials at all stages of CKD.^5^ In addition, new disease-modifying therapies for CKD that slow progression are available, but they have been largely studied in patients with preserved kidney function.^6^ The optimal use of these therapies is in high-risk individuals with early stages of CKD where the benefit for dialysis prevention is large and cost-effectiveness may be optimal.^7^

Accurate models to predict a 40% decline in eGFR or the composite outcome of kidney failure or 40% decline in eGFR that can be applied to patients at all stages of CKD (G1–G5) are needed. When these models are based on laboratory data, they can be used through electronic health records or laboratory information systems, and are not subject to variability in coding, often found with CKD and its complications. We present here the derivation and external validation of new laboratory-based machine learning prediction models that accurately predict 40% decline in eGFR or kidney failure in patients with CKD G1 to G5.

## Methods

### Study Population

#### Development Cohort

The development cohort was derived from administrative data in Manitoba, Canada (population 1.4 million), using data from the Manitoba Centre for Health Policy. The Manitoba Centre for Health Policy is a research unit within the Department of Community Health Sciences at the University of Manitoba that maintains a population-based repository of data on health services and other social determinants of health covering all individuals in the province. We identified all adult (age 18+ years) individuals in the province with an available outpatient eGFR test between April 1, 2006, and December 31, 2016, with valid Manitoba Health registration for at least 1-year preindex. eGFR was calculated from available serum creatinine tests using the CKD-Epidemiology Collaboration equation.^8^ Included patients were further required to have complete demographic information on age and sex, including the result of at least 1 urine ACR or protein-to-creatinine ratio (PCR) test. Patients with a history of kidney failure (dialysis or transplant) were excluded.

#### Validation Cohort

The validation cohort was derived from the Alberta Health database. This database contains information on demographic data, laboratory data, hospitalizations, and physician claims for all patients in the province of Alberta, Canada (population 4.4 million). Regular laboratory coverage for creatinine measurements and ACR/PCR values is complete from 2005; however, additional laboratory values are fully covered only from 2009 onward. As such, we identified a cohort of individuals with at least 1 calculable eGFR, valid health registration, and an ACR (or imputed PCRs) value starting from April 1, 2009, to December 31, 2016. We randomly sampled one-third of the external cohort to perform the final analysis to reduce computation time. Patients with a history of kidney failure (dialysis or transplant) were excluded.

This study was reviewed and approved by the institutional ethics review board at the Universities of Manitoba (Health Research Ethics Board—Ethics #HS21776:H2018:179) Alberta (Pro00053469), and Calgary (REB16-1575). Informed consent was not required as all data were provided deidentified using a scrambled personal health information number.

### Variables

#### Independent Variables

All models included age, sex, eGFR, and urine ACR as described previously. Baseline eGFR was calculated as the average of all available outpatient eGFR results beginning with the first recorded eGFR during the study period and moving forward to the last available test in a 6-month window and calculating the mean of tests during this period. The index date of the patient was considered the date of the final eGFR in this 6-month period.^9^ Age was determined as the date of the index eGFR, and sex was determined using a linkage to the Manitoba Health Insurance Registry which contained dates of birth and other demographic data. If a urine ACR test was unavailable, we converted available urine PCR tests to corresponding urine ACRs using published and validated equations.^10^ The closest result within 1 year before or after the index date was selected. Urine ACR was log transformed to handle the skewed distribution as in previous studies.

In addition to the previously described variables, we also evaluated the utility of additional laboratory results from chemistry panels, liver enzymes, and complete blood cell count panels for inclusion in the random forest model based on associations found in previous studies of models predicting CKD progression.^11^, ^12^, ^13^ The closest value within 1 year of the index date was selected (before or after) for inclusion. Distributional transformations were applied when needed. The final random forest model included eGFR, urine ACR, and an additional 18 laboratory results. An overview of the degree of missingness for the laboratory panels is provided in Supplementary Table S1. The random forest models applied imputations for missing data using the method of Ishwaran *et al.*^14^

All laboratory data included were extracted from the Shared Health Diagnostic Services of Manitoba Laboratory Information System, and any values recorded during a hospitalization event as determined by a linkage to the Discharge Abstract Database were not included (inpatient tests). For the validation cohort, Alberta Health laboratory data were extracted from the Alberta Kidney Disease Network. Of the 18 laboratory tests used in the Manitoba model, 16 were also regularly collected by the Alberta Kidney Disease Network. The unavailable tests (aspartate aminotransferase and gamma glutamyl transferase) were treated as missing data.

#### Dependent Variable—Development Cohort

The primary outcome in our study was a 40% decline in eGFR or kidney failure. The 40% decline in eGFR was determined as the first eGFR test in the laboratory data that was 40% or greater in decline from the baseline eGFR, requiring a second confirmatory test result between 90 days and 2 years after the first test unless the patient dies or experiences kidney failure within 90 days after the first test result revealing a 40% or greater decline. Therefore, a patient experiencing a single eGFR representing a 40% decline and dying within 90 days is treated as an event, or if they experience kidney failure in that period.^15^ Kidney failure was defined as initiation of chronic dialysis, receipt of a transplant, or an eGFR <10 ml/min per 1.73 m^2^. Dialysis was defined as any 2 claims in the Manitoba Medical Services database for chronic dialysis, and transplant was defined as any 1 claim in the Manitoba Medical Services database for kidney transplant or a hospitalization in the Discharge Abstract Database with a corresponding procedure code for kidney transplantation (1PC85 or 1OK85 using the Canadian Classification of Health Interventions codes or International Classification of Diseases, Ninth Revision, procedure code 55.6). An overview of tariff codes identifying dialysis and transplant is provided in Supplementary Table S2. The outcome date for the 40% decline in eGFR or kidney failure was determined based on the first of these events.^6^ Patients were followed until reaching the above-mentioned composite end point, death (as determined by a linkage to the Manitoba Health Insurance Registry), a maximum of 5 years, or loss to follow-up.

#### Dependent Variable—Validation Cohort

Using laboratory creatinine measurements as described for the Manitoba cohort described previously, 40% decline in eGFR was identified. Kidney failure was defined similarly, but with minor adaptations necessitated by a structurally different administrative data set (Supplementary Table S2). Chronic dialysis and kidney transplants were identified using the Northern and Southern Alberta Renal Program databases, a provincial registry of renal replacement—any single code for hemodialysis, peritoneal dialysis, or transplant was used. (Note: Because the registry begins in 2001, physician claims data were also used when excluding individuals with prior transplants or dialysis). We linked these data sources to the provincial laboratory repository by unique, encoded, patient identifiers.

### Statistical Analysis

Baseline characteristics for the development (internal training and testing) and external validation cohorts were summarized with descriptive statistics. We developed a random forest model using the R package Fast Unified Random Forest for Survival, Regression, and Classification using a survival forest with right-censored data.^16^ Data were split into training (70%) and testing (30%) data sets with a single split and then validated in an external cohort. Models were evaluated for accuracy using the area under the receiver operating characteristic curve, the Brier score, and calibration plots of observed versus predicted risk. Area under the receiver operating characteristic curve and Brier scores were assessed for prediction of the outcome at 1 to 5 years, in 1-year intervals, and calibration plots were evaluated at 2 and 5 years. Model hyperparameters were optimized using the *tune.rfsrc* function using comparisons of the maximal size of the terminal node and the number of variables to possibly split at each node to the out-of-bag error rate from the Random Forest for Survival, Regression, and Classification package.^16^

In addition, we assessed sensitivity, specificity, negative predictive value (NPV), and positive predictive value (PPV) for the top 10%, 15%, and 20% of patients at highest estimated risk (high risk), including for the bottom 50%, 45%, and 30% at lowest risk (low risk). These metrics were assessed at 2 and 5 years. A visualization of the risk of progression versus predicted probability was plotted for 2 and 5 years. Using the final grown 22-variable forest, variable importance of included parameters was evaluated, with results for the 5 most influential variables presented.^17^

To evaluate robustness, we evaluated the model in subpopulations of the testing and validation cohorts for the 5-year prediction of the primary outcome defined by CKD stage and the presence or absence of diabetes.

### Sensitivity Analyses

For sensitivity analyses, we considered 2 comparator models. (i) We evaluated a Cox proportional hazards model using a guideline-based definition of risk using the 3-level definition of albuminuria and 5 stages of eGFR as categorical predictors as a comparator (heatmap model).^18^ (ii) We evaluated a Cox proportional hazards model including the variables eGFR, urine ACR, diabetes, hypertension, stroke, myocardial infarction, age, and sex (clinical model). In addition, we evaluated the model in the external validation cohort where laboratory values were only included 1 year before the index date.

Analysis was performed using R Version 4.1.0. Statistical significance was a priori identified using an α = 0.05.

## Results

### Cohort Selection

For the development cohort (training and testing), we had a total sample size of 77,196, allocating 54,037 to the training data set (70%) and 23,159 to the testing data set. A total of 321,396 individuals were identified in the validation cohort, with a random subset of 107,097 selected for evaluation. Detailed overview of the cohort selection process for both the development and validation cohorts is provided in Supplementary Figure S1.

### Cohort Description

The mean age of the development cohort was 59.3 years, with a mean eGFR of 82.2 ml/min per 1.73 m^2^ and median urine ACR of 1.1 mg/mmol. Of the patients, 48% were male, 45% had diabetes, 70% had hypertension, 5% had a history of congestive heart failure, 4% a prior stroke, and 3% a prior myocardial infarction (similar between the testing and training cohorts). The validation cohort was slightly younger, with a mean age of 55.5 years, mean eGFR of 86.0 ml/min per 1.73 m^2^, and median ACR of 0.8 mg/mmol. The validation cohort had a higher proportion of male patients (53%), 41% of patients had diabetes, 51% hypertension, 5% a history of congestive heart failure, 5% a prior stroke, and 5% a prior myocardial infarction. An overview of baseline descriptive statistics is provided in Table 1.

**Table 1:** Baseline characteristics of development and validation cohorts

Clinical characteristics	Training (Manitoba) *n* = 54,037	Internal testing (Manitoba) *n* = 23,159	External validation (Alberta) *n* = 107,097
Age	59.3 (17)	59.3 (17.1)	55.5 (16.2)
Male sex	25,829 (48%)	11,017 (48%)	57,168 (53%)
eGFR	82.2 (27.1)	82.2 (27.3)	86.0 (22.4)
Urine ACR (mg/mmol)	1.1 (0.5–4.7)	1.1 (0.5–4.7)	0.8 (0.4–2.2)
Comorbid conditions			
Diabetes	24,460 (45%)	10,428 (45%)	43,504 (41%)
Hypertension	37,701 (70%)	16,275 (70%)	54,637 (51%)
Congestive heart failure	2840 (5%)	1187 (5%)	5808 (5%)
Prior stroke	1937 (4%)	832 (4%)	5590 (5%)
MI	1380 (3%)	608 (3%)	6382 (6%)
Laboratory characteristics			
Urea (mmol/l)	6.6 (4.0)	6.6 (4.1)	6.8 (3.9)
Serum hemoglobin (g/l)	134 (19)	133 (19)	143 (16)
Glucose (mmol/l)	7.9 (4.1)	7.9 (4.1)	7.4 (4.1)
Serum albumin (g/l)	37 (6)	37 (6)	41 (4)
Events (5 yr)			
40% decline	3965 (7.3%)	1658 (7.2%)	5106 (4.8%)
Kidney failure	246 (0.5%)	102 (0.4%)	367 (0.3%)
Composite	4211 (7.8%)	1760 (7.6%)	5473 (5.1%)

ACR, albumin-to-creatinine ratio; eGFR, estimated glomerular filtration rate; MI, myocardial infarction.

### Model Performance in Internal Testing Cohort

In the random forest model with 22 variables, when evaluated in the testing cohort, we found an AUC of 0.90 (0.89–0.92) for 1-year prediction of the primary outcome and 0.84 (0.83–0.85) for 5-year prediction. The Brier score was 0.02 (0.01–0.02) for 1-year prediction of the primary outcome and 0.07 (0.06–0.09) for 5-year prediction. AUCs and Brier scores for years 1 to 5 are presented in Table 2. AUC and Brier score were similar in the predefined subgroups (Supplementary Table S3).

**Table 2:** Results of the 22-variable random forest model in the internal testing and external validation cohorts for prediction of 40% decline in eGFR or kidney failure

Time frame, yr	Internal testing cohort (Manitoba) *n* = 23,159		External validation cohort (Alberta) *n* = 107,097	
	AUC (95% CI)	Brier score (95% CI)	AUC (95% CI)	Brier score (95% CI)
1	0.90 (0.89–0.92)	0.02 (0.01–0.02)	0.87 (0.86–0.89)	0.01 (0.01–0.01)
2	0.88 (0.87–0.89)	0.03 (0.03–0.04)	0.87 (0.86–0.88)	0.01 (0.01–0.01)
3	0.86 (0.85–0.87)	0.05 (0.04–0.06)	0.86 (0.85–0.86)	0.02 (0.02–0.02)
4	0.85 (0.84–0.86)	0.06 (0.05–0.07)	0.85 (0.84–0.86)	0.03 (0.03–0.03)
5	0.84 (0.83–0.85)	0.07 (0.06–0.09)	0.84 (0.84–0.85)	0.04 (0.04–0.04)

ACR, albumin-to-creatinine ratio; eGFR, estimated glomerular filtration rate.

**Figure 1:**
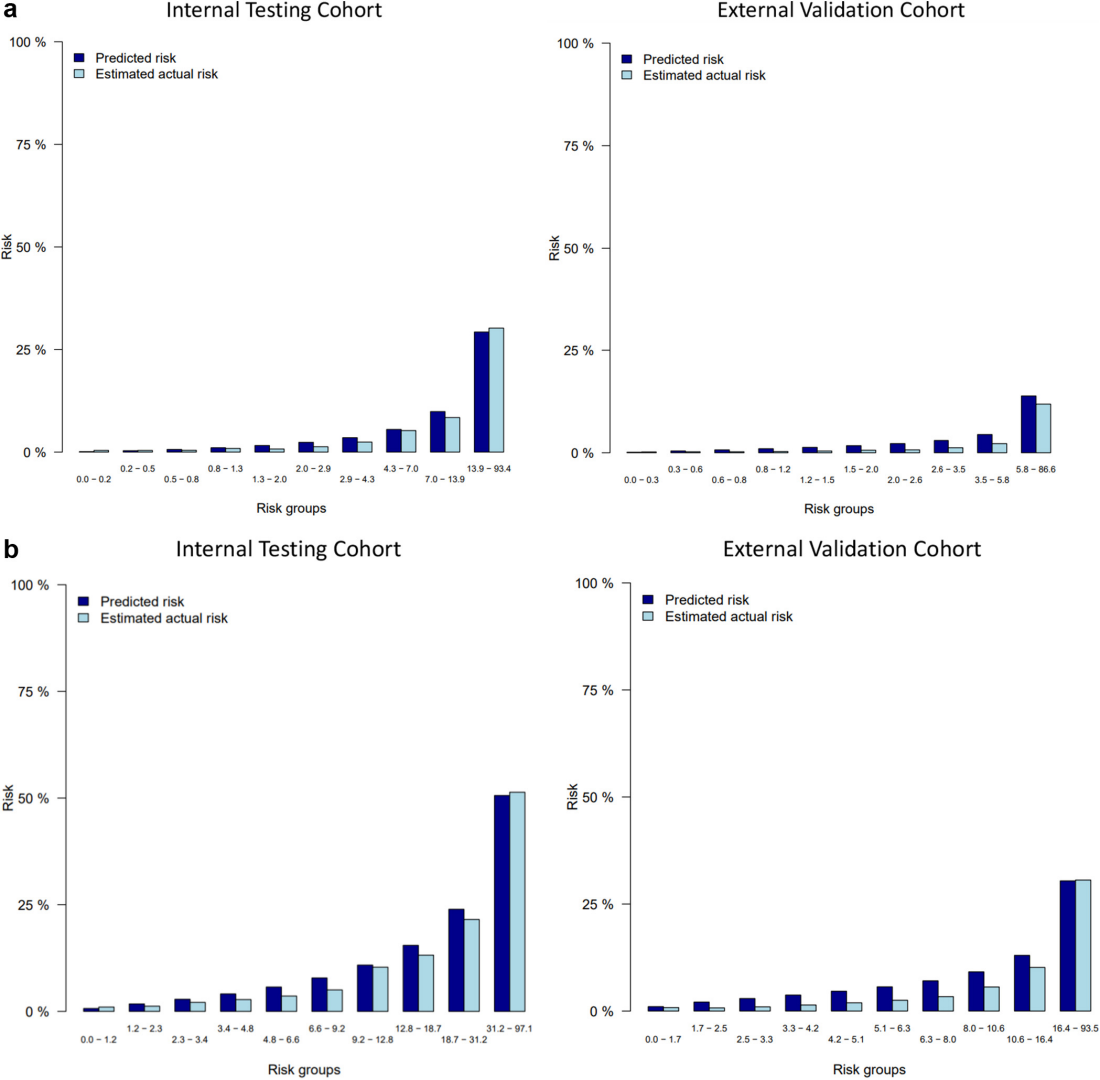
Calibration for the 22-variable random forest model for prediction of 40% decline in eGFR or kidney failure at (a) 2 years and at (b) 5 years. eGFR, estimated glomerular filtration rate.

**Figure 2:**
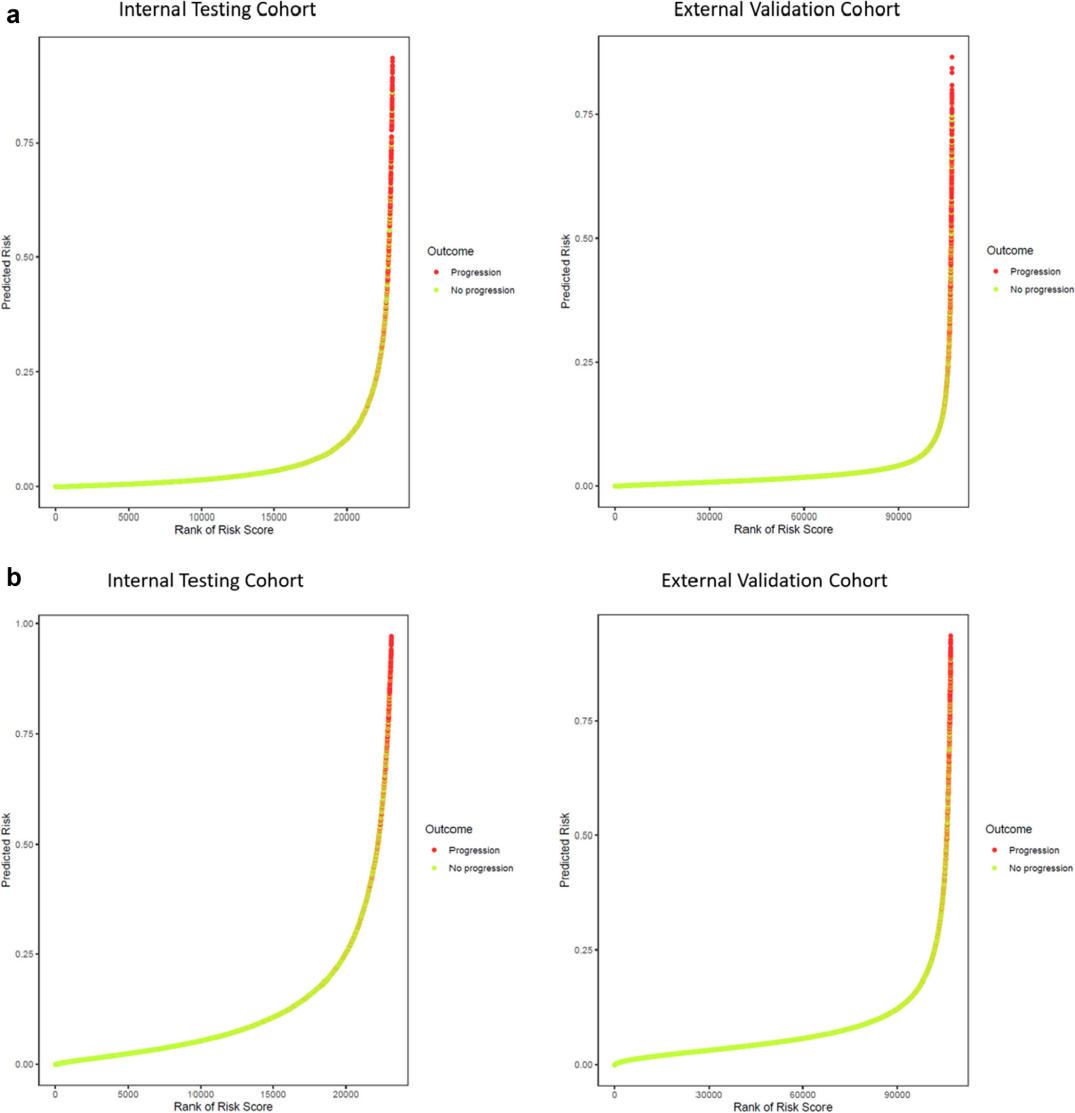
Relationship between predicted risk from the random forest algorithm and the occurrence of the primary outcome (40% decline in eGFR or kidney failure) at (a) 2 years and at (b) 5 years. eGFR, estimated glomerular filtration rate.

We observed excellent calibration at both 2 and 5 years (Figures 1a) in both the internal and external testing cohorts. In addition, a relationship between occurrence of the primary outcome event was observed to increase with increasing predicted probability generated by the random forest algorithm (Figures 2a).

We evaluated statistics on sensitivity, specificity, and PPV in high-risk patients (top 10%, 15%, and 20% of risk scores, respectively). For prediction of the primary outcome at 2 years, we found that patients in the top decile (14% 2-year risk threshold) had a sensitivity of 58%, a specificity of 92%, and a PPV of 25%. Similarly, for the top 15% of patients (10% 2-year risk threshold), we found a sensitivity of 69%, specificity of 87%, and PPV of 20%. For the top 20% of patients (7% 2-year risk threshold) sensitivity was 76%, specificity was 83%, and PPV was 16%. Using a 30% threshold to identify high- and intermediate-risk patients, we would have identified 87% of individuals with an event in 2 years and 77% within 5 years.

In the low-risk patients, we found that the bottom 50% of patients (1.95% 2-year risk threshold) had a sensitivity of 94%, specificity of 52%, and NPV of >99%. For the lowest 45% of risk scores (1.61% 2-year risk threshold), sensitivity was 95%, specificity was 47%, and NPV was >99%. Last, for the lowest 30% of risk scores (0.85% 2-year risk threshold), we found a sensitivity of 97%, a specificity of 31%, and an NPV >99%. We also considered these statistics for the prediction of the outcome at 5 years and found similar accuracy (Table 3).

**Table 3:** Overview of model performance for the 22-variable random forest model

2 Yr Threshold Low risk	Internal testing cohort (*n* = 23,159)				2 Yr Predicted risk Low risk	External validation cohort (*n* = 107,097)			
	Population	Sens	Spec	NPV/PPV NPV		Population	Sens	Spec	NPV/PPV NPV
0.85	Lowest 30	97	31	>99	0.84	Lowest 30	96	31	>99
1.61	Lowest 45	95	47	>99	1.31	Lowest 45	93	46	>99
1.95	Lowest 50	94	52	>99	1.51	Lowest 50	91	51	>99


High risk				PPV	High risk				PPV
7	Top 20	76	83	16	4	Top 20	77	81	7
10	Top 15	69	87	20	4	Top 15	73	86	8
14	Top 10	58	92	25	6	Top 10	65	91	11


5 Yr	Internal testing cohort (*n* = 23,159)				5 Yr	External validation cohort (*n* = 107,097)			
Threshold Low risk	Population	Sens	Spec	NPV/PPV NPV	Predicted risk Low risk	Population	Sens	Spec	NPV/PPV NPV
3.40	Lowest 30	97	32	99	3.33	Lowest 30	95	31	99
5.70	Lowest 45	93	48	99	4.61	Lowest 45	91	47	99
6.70	Lowest 50	91	53	99	5.11	Lowest 50	89	52	99

High risk				PPV	High risk				PPV
19	Top 20	67	84	25	11	Top 20	69	83	18
24	Top 15	59	89	30	13	Top 15	61	88	21
31	Top 10	48	93	36	17	Top 10	50	92	26

NPV, negative predictive value; PPV, positive predictive value; Sens, sensitivity; Spec, specificity.

Data presented in percentage.

Urine ACR (including converted PCRs) was the most influential variable in the random forest model, followed by eGFR, urea, hemoglobin, age, serum albumin, hematocrit, and glucose. An overview of model inputs ranked by importance is detailed in Supplementary Table S4.

### Model Performance in External Validation

Performance was found to be similar when evaluated in the external validation cohort with an AUC of 0.87 (0.86–0.89) for 1-year prediction declining to 0.84 (0.84–0.85) for 5-year prediction, with Brier scores of 0.01 (0.01–0.01) at 1 year and 0.04 (0.04–0.04) at 5 years (Table 2). The external validation cohort had a lower overall risk at both 2 years and 5 years, but we found excellent calibration (Figure 1b) and a similar association between rank of the risk score and probability of the composite outcome (Figure 2b). In addition, subgroup analyses in patients with and without diabetes, CKD stages G1 to G3, and eGFR <60 ml/min per 1.73 m^2^ had similar outcomes to the internal testing cohort (Supplementary Table S3). Similar diagnostic accuracy, evaluated with sensitivity, specificity, NPV, and PPV, was observed in the external validation cohort as that of the development cohort (Table 3).

### Sensitivity Analyses

In the comparator analysis, the heatmap model performed worse than the 22-variable random forest model in the development cohort (C statistic 0.78 at 5 years vs. 0.84, Supplementary Table S5), as did the clinical model (C statistic 0.81 at 5 years, *P* < 0.001, Supplementary Table S6). When considering only laboratory values in the 12 months preceding the index date, the results of model evaluation for the random forest model were unchanged (1-year AUC of 0.87, 0.86–0.88; 5-year AUC 0.84, 0.83–0.85).

## Discussion

In this retrospective cohort study of >177,000 individuals from a public health care system, we developed and externally evaluated laboratory-based prediction models for the outcomes of kidney failure or 40% decline in eGFR. Our models are entirely based on a single time point measure of routinely collected laboratory data and predict the outcomes of interest (CKD progression) with greater accuracy than current standard of care or commercially available models that test for novel biomarkers and use machine learning methods.^19^ Taken together, our novel model can be implemented in clinical and research settings.

Previous investigators have developed prediction models for the progression of CKD to kidney failure and for intermediate outcomes that include incident CKD, rapid decline in kidney function, or 40% decline in eGFR.^19^ Despite adequate sample sizes and evidence of internal validity, most of these models have not been externally validated nor translated into routine clinical practice. In contrast, the Kidney Failure Risk Equations developed in 2011,^11^ and internationally validated in 2016,^20^ have been translated into clinical practice guidelines, electronic health records, and laboratory information systems and are used to guide access to nephrology referral, case management, and interdisciplinary care in many jurisdictions.^21^ Although the Kidney Failure Risk Equations have similar accuracy to the other published models, it is likely the usability/simplicity of the equations and their reliance on laboratory data have driven their adoption. With this in mind, we sought to develop these new models for CKD progression focusing primarily on laboratory data sources and only adding demographic variables and comorbid conditions if they meaningfully improved discrimination or calibration.

Our machine models using a random forest seem to perform better than a commercially available machine learning model (RenalytixAI).^19^ Compared with the RenalytixAI tool, our model has the advantage of having had external validity in an independent population and is therefore at lower risk for overfitting. This step is particularly important for machine learning models which, when derived in small data sets with many predictors, tend to overfit the development population and often do not generalize well. Our models require only easily mapped laboratory data, which may make them easier to implement at scale than models requiring multiple electronic health record fields and data types, such as the RenalytixAI tool. Finally, our test does not require the measurement of 3 novel biomarkers as does RenalytixAI, and therefore can be performed in a routine laboratory setting or using already collected laboratory data.

There are important clinical and research implications of our proposed models: From a clinical perspective, physicians can use our tool in the office to identify patients who are early in their course of CKD (eGFR >60 ml/min per 1.73 m^2^), but at high risk of progression in the next 5 years. Given the effect of interventions such as SGLT2i on the slope of eGFR in this population, it is possible that these patients may be able to forestall or prevent the lifetime occurrence of kidney failure entirely versus delaying the time to dialysis if the interventions are implemented later in course of disease.^22^ In addition, newer therapies such as finerenone may provide additional benefit for slowing CKD progression; however, they have been largely studied in patients with preserved kidney function and may be initially reserved for intermediate and high risk subgroups to maximize benefit while reducing the burden of cost and polypharmacy.^23^ From a research perspective, several large clinical trials have used 40% decline in eGFR or kidney failure as the primary outcome, and validation of our models in those trial data sets may help highlight risk treatment interactions. For future trials that are currently in planning or enrolment phases, the use of our models may be helpful to enrich the trial population to generate the appropriate number of outcomes in a reasonable time frame.

Strengths of our analysis include external validation, which is particularly important for machine learning models as they can overfit small data sets that have many predictor variables. In addition to this point, we found that the model was able to externally validate with high discrimination in a cohort that had total missingness for 2 variables. Additional strengths include novel research methods that include random forest methodology on 2 well described data sets, findings from which have been proven generalizable for multiple kidney outcomes and interventions. A notable strength is the reliance only on routinely collected laboratory data, enabling rapid integration into electronic health records and laboratory information systems.

There are also limitations to consider. First, the model was developed and validated in a Canadian population and requires further validation in other settings. Second, the model includes individuals with urine protein quantification and requires further validation in settings where this information may not be available. Third, we currently lack an online calculator or an electronic health record/laboratory information system integration, which would help broader adoption. Last, future research needs to be conducted to consider the cost-effectiveness and efficacy/safety of a model guided strategy to determine the prescription of disease-modifying therapy.

In conclusion, we present new models that use routinely collected laboratory data and predict CKD progression (40% decline in eGFR or kidney failure) with accuracy for all patients with CKD. These models can have important clinical and research benefits and should be further externally validated and implemented in health care settings.

## Disclosure

NT reports receiving grants, personal fees, and other from Tricida Inc.; grants and personal fees from Astra Zeneca Inc., Janssen, and Bayer; personal fees from Otsuka Inc., Boehringer Ingelheim/Eli Lilly, and Roche; other from PulseData and Mesentech; personal fees and other from Renibus, outside the submitted work; serving as the founder of Klinrisk and Clinpredict Inc., in which Klinrisk develops models for CKD progression and Clinpredict works on implementation of models in electronic health records; serving as scientific advisor to PulseData Inc., in which PulseData develops customized machine models for adverse outcomes in kidney disease. TF reports receiving personal fees from Strategic Health Resources, Quanta Dialysis Technologies Ltd., and Baxter Canada outside the submitted work; and personal fees from Clinpredict Inc. MMS received speaker’s fees from AstraZeneca outside the submitted work. PK reports receiving personal fees and other from Quanta Dialysis Technologies Ltd. outside the submitted work. CR reports receiving personal fees from Otsuka and Bayer and personal fees and other from Health Logic Interactive outside the submitted work.

## Supplementary Material

Supplementary File (PDF)

**Figure S1.** Overview of cohort selection.

**Table S1.** Overview of variable missingness in internal testing and external validation cohorts.

**Table S2.** Definition of dialysis and transplantation. NARP, Northern Alberta Renal Health Program; SARP, Southern Alberta Renal Health Program; CCP, Canadian Classification of Diagnostic, Therapeutic, and Surgical Procedures; AHIP, Alberta Health Care Insurance Plan.

**Table S****4****.** Overview of variable importance by random branch assignment for the 8 most influential parameters in the random forest model.

**Table S****3****.** Results of subgroup analyses for prediction of 40% decline in eGFR or kidney failure at 5 years.

**Table S5.** Results of guideline-based model (heatmap model).

**Table S6.** Results of clinical model.

## References

[1] Coresh J., Selvin E., Stevens L.A. (2007). Prevalence of chronic kidney disease in the United States. J Am Med Assoc.

[2] Golestaneh L., Alvarez P.J., Reaven N.L. (2017). All-cause costs increase exponentially with increased chronic kidney disease stage. Am J Manag Care.

[3] Smekal M.D., Tam-Tham H., Finlay J. (2019). Patient and provider experience and perspectives of a risk-based approach to multidisciplinary chronic kidney disease care: a mixed methods study. BMC Nephrol.

[4] Nelson J., Kent D.M., Dahabreh I.J. (2016). Risk and treatment effect heterogeneity: re-analysis of individual participant data from 32 large clinical trials. Int J Epidemiol.

[5] Inker L.A., Lambers Heerspink H.J., Mondal H. (2014). GFR decline as an alternative end point to kidney failure in clinical trials: a meta-analysis of treatment effects from 37 randomized trials. Am J Kidney Dis.

[6] Bakris G.L., Agarwal R., Anker S.D. (2020). Effect of finerenone on chronic kidney disease outcomes in type 2 diabetes. N Engl J Med.

[7] Komenda P., Ferguson T.W., Macdonald K. (2014). Cost-effectiveness of primary screening for CKD: a systematic review. Am J Kidney Dis.

[8] Levey A.S., Stevens L.A., Schmid C.H. (2009). A new equation to estimate glomerular filtration rate. Ann Intern Med.

[9] Hemmelgarn B.R., Clement F., Manns B.J. (2009). Overview of the Alberta Kidney Disease Network. BMC Nephrol.

[10] Weaver R.G., James M.T., Ravani P. (2020). Estimating urine albumin-to-creatinine ratio from protein-to-creatinine ratio: development of equations using same-day measurements. J Am Soc Nephrol.

[11] Tangri N., Stevens L.A., Griffith J. (2011). A predictive model for progression of chronic kidney disease to kidney failure. JAMA.

[12] Chang H.L., Wu C.C., Lee S.P., Chen Y.K., Su W., Su S.L. (2019). A predictive model for progression of CKD. Medicine (Baltimore).

[13] Zacharias H.U., Altenbuchinger M., Schultheiss U.T. (2022). A predictive model for progression of CKD to kidney failure based on routine laboratory tests. Am J Kidney Dis.

[14] Ishwaran H., Kogalur U.B., Blackstone E.H., Lauer M.S. (2008). Random survival forests. Ann Appl Stat.

[15] Nelson R.G., Grams M.E., Ballew S.H. (2019). Development of risk prediction equations for incident chronic kidney disease. JAMA.

[16] Ishwaran H., Kogalur U.B. RandomForestSRC. Random forests for survival, regression and classification (RF-SRC). https://cran.r-project.org/web/packages/randomForestSRC/randomForestSRC.pdf.

[17] Ishwaran H. (2007). Variable importance in binary regression trees and forests. Electron J Statist.

[18] Levey A.S., De Jong P.E., Coresh J. (2011). The definition, classification, and prognosis of chronic kidney disease: a KDIGO Controversies Conference report. Kidney Int.

[19] Chan L., Nadkarni G.N., Fleming F. (2021). Derivation and validation of a machine learning risk score using biomarker and electronic patient data to predict rapid progression of diabetic kidney disease. Diabetologia.

[20] Tangri N., Grams M.E., Levey A.S. (2016). Multinational assessment of accuracy of equations for predicting risk of kidney failure a meta-analysis. JAMA.

[21] Hingwala J., Wojciechowski P., Hiebert B. (2017). Risk-based triage for nephrology referrals using the kidney failure risk equation. Can J Kidney Heal Dis.

[22] Perkovic V., Jardine M.J., Neal B. (2019). Canagliflozin and renal outcomes in type 2 diabetes and nephropathy. N Engl J Med.

[23] Bakris G.L., Agarwal R., Anker S.D. (2019). Design and baseline characteristics of the finerenone in reducing kidney failure and disease progression in diabetic kidney disease trial. Am J Nephrol.

