# Supplementary material for: Development and External Validation of a Machine Learning Model for Progression of CKD
Source: Kidney Int Rep. 2022 May 13;7(8):1772–81. doi: 10.1016/j.ekir.2022.05.004 (PMC9366291; doi:10.1016/j.ekir.2022.05.004)
Supplement: Supplementary File (PDF) [file mmc1.pdf]

Appendix Figure S1. Overview of Cohort Selection

Development Cohort (Manitoba)

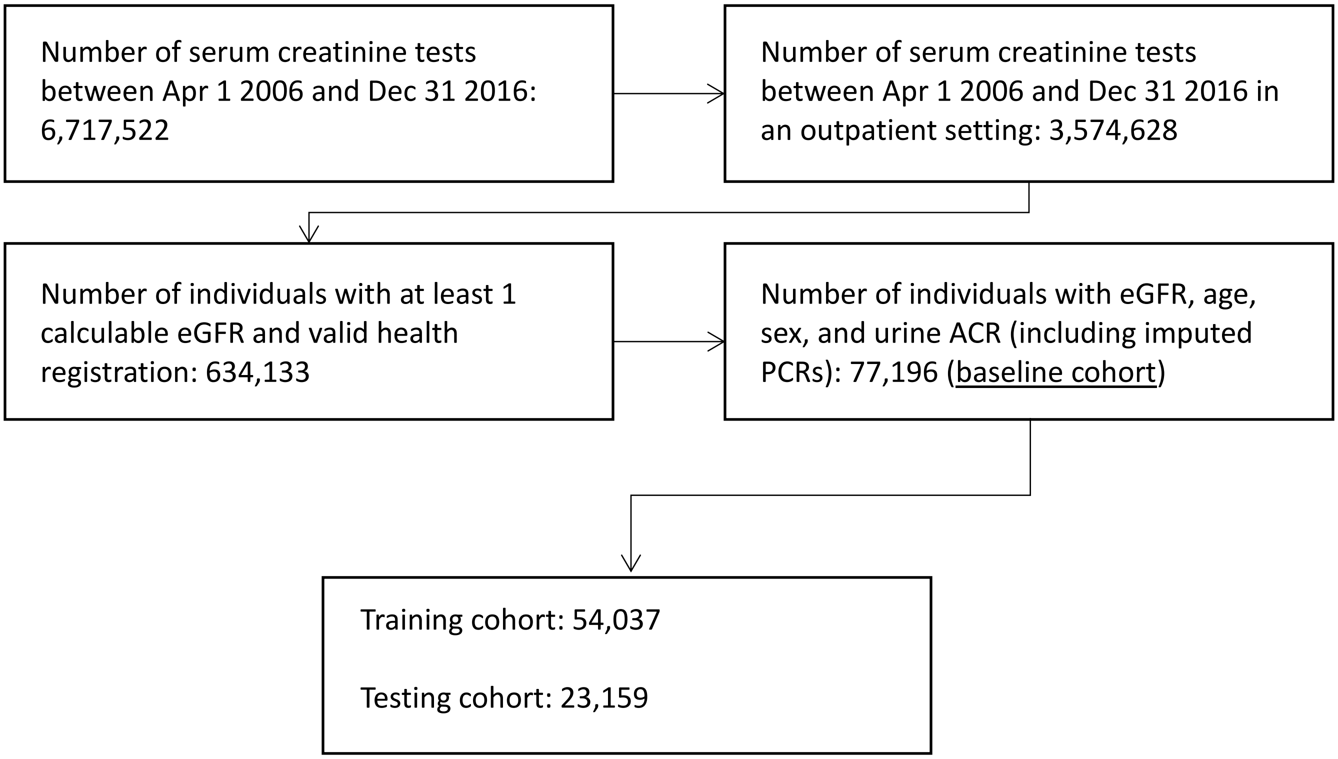

Validation Cohort (Alberta)

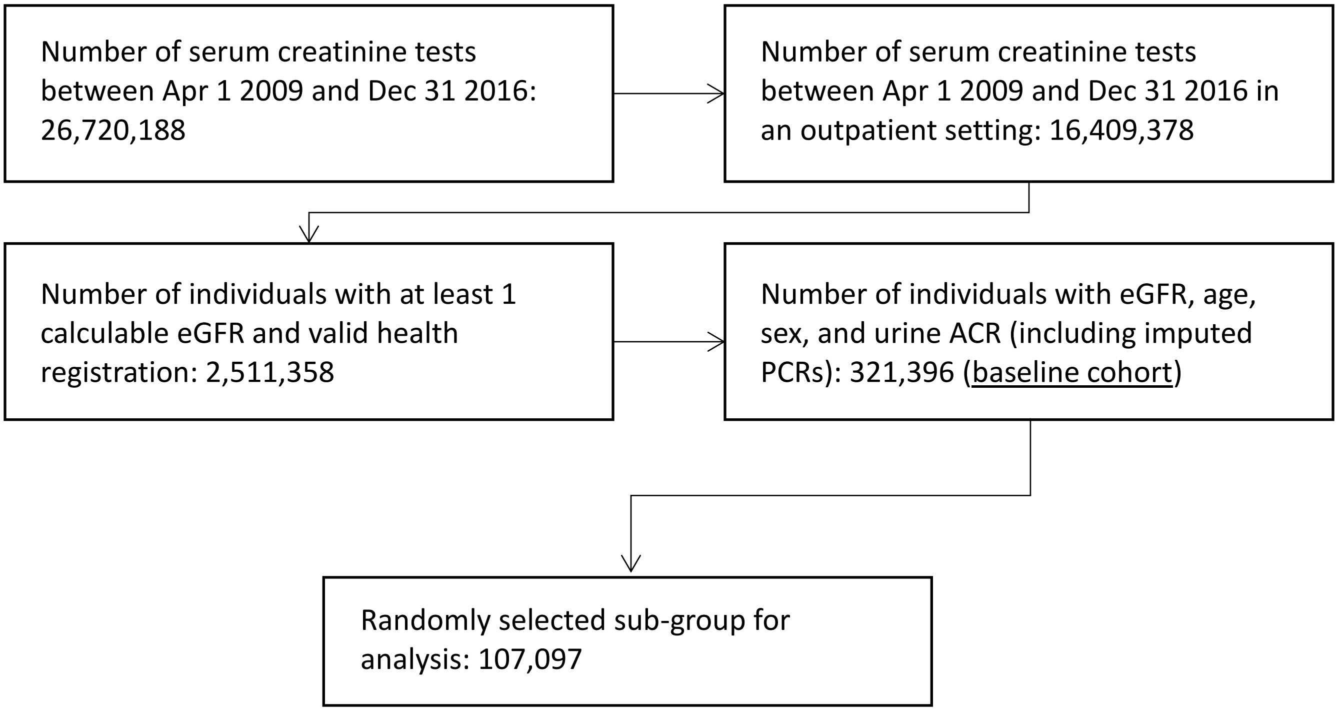

Appendix Table S1. Overview of Variable Missingness in Internal Testing and External Validation Cohorts

	Percent Missing	
**Variable**	**Manitoba**	**Alberta**
Age	0%	0%
eGFR	0%	0%
Urine ACR	0%	0%
Sex	0%	0%
Chemistry Panel	5 - 59%	11 - 78%
Liver Enzymes	36 - 49%	13 - 100%
Complete Blood Panel	9%	3 - 9%

Appendix Table S2. Definition of Dialysis and Transplantation

Manitoba Cohort

Item	Manitoba Health Tariff Codes
Dialysis (as validated in Komenda, Paul, et al. "Determination of the optimal case definition for the diagnosis of end-stage renal disease from administrative claims data in Manitoba, Canada." *CMAJ open* 3.3 (2015): E264.)	Initial dialysis (9798, 9801, 9805, 9806), subsequent dialysis (9799, 9802, 9807, 9819) and home dialysis (9821)
Transplantation	5883

Alberta Cohort

Item	Dataset	Definition
Maintenance dialysis (hemodialysis, peritoneal dialysis)	NARP, SARP (2002 – 2018)	At least 2 physician claims for any of the dialysis procedure codes:<br>AHCIP: 13.99A, 13.99B, 13.99C, 13.99D, 13.99O, 13.99OA
	AH (1994 – 2002)	
Kidney transplantation	NARP, SARP (2002 – 2018)	ICD-9-CM: 5569<br>CCP: 67.59
	AH (1994 – 2002)	

NARP: Northern Alberta Renal Health Program

SARP: Southern Alberta Renal Health Program

CCP: Canadian Classification of Diagnostic, Therapeutic, and Surgical Procedures

AHIP: Alberta Health Care Insurance Plan

Appendix Table S3. Results of Subgroup Analyses for Prediction of 40% Decline in eGFR or Kidney Failure at 5 Years

	Internal Testing Cohort (Manitoba)			External Validation Cohort (Alberta)		
**Subgroup**	**n**	**AUC**	**Brier**	**n**	**AUC**	**Brier**
Total Testing Cohort	23,159	0.84 (0.83 – 0.85)	0.08 (0.06 – 0.09)	107,097	0.84 (0.84 – 0.85)	0.04 (0.04 – 0.04)
Patients with diabetes	10,428	0.82 (0.80 – 0.84)	0.09 (0.09 – 0.10)	43,504	0.82 (0.81 – 0.83)	0.07 (0.07 – 0.07)
Patients without diabetes	12,731	0.85 (0.83 – 0.86)	0.06 (0.05 – 0.06)	63,593	0.84 (0.83 – 0.85)	0.03 (0.02 – 0.03)
CKD Stages G1 - G3	8,620	0.77 (0.76 – 0.79)	0.12 (0.11 – 0.13)	27,706	0.77 (0.76 – 0.77)	0.10 (0.09 – 0.10)
CKD Stages G3 - G5	4,807	0.81 (0.79 – 0.82)	0.14 (0.13 – 0.15)	13,705	0.80 (0.79 – 0.81)	0.12 (0.12 – 0.13)

Appendix Table S4. Overview of Variable Importance by Random Branch Assignment for the 5 Most Influential Parameters in the Random Forest Model

Parameter	Variable Importance
urine ACR	0.048605
eGFR	0.024517
urea	0.019901
hemoglobin	0.013356
age	0.00647
albumin	0.006205
hematocrit	0.0056
glucose	0.002923

Appendix Table S5. Results of guideline-based model (heatmap model)

	Internal Testing Cohort (Manitoba) n = 23,159		
**Timeframe**	**AUC (95% CI)**	**Decrease from Random Forest Model (95% CI)**	**p-value**
1 year	0.84 (0.82 – 0.86)	0.07 (0.05 – 0.08)	<0.001
2 years	0.82 (0.81 – 0.83)	0.06 (0.05 – 0.07)	<0.001
3 years	0.80 (0.79 – 0.82)	0.06 (0.05 – 0.07)	<0.001
4 years	0.79 (0.78 – 0.80)	0.06 (0.05 – 0.07)	<0.001
5 years	0.78 (0.77 – 0.79)	0.06 (0.05 – 0.06)	<0.001

	Internal Testing Cohort (Manitoba) n = 23,159		
**Timeframe**	**Brier Score (95% CI)**	**Increase from Random Forest Model (95% CI)**	**p-value**
1 year	0.02 (0.02 – 0.02)	0.002 (0.002 – 0.003)	<0.001
2 years	0.04 (0.04 – 0.04)	0.004 (0.003 – 0.005)	<0.001
3 years	0.05 (0.05 – 0.06)	0.005 (0.004 – 0.006)	<0.001
4 years	0.07 (0.06 – 0.07)	0.006 (0.005 – 0.007)	<0.001
5 years	0.08 (0.08 – 0.09)	0.007 (0.006 – 0.008)	<0.001

Appendix Table S6. Results of clinical model

	Internal Testing Cohort (Manitoba) n = 23,159		
**Timeframe**	**AUC (95% CI)**	**Decrease from Random Forest Model (95% CI)**	**p-value**
1 year	0.85 (0.83 – 0.86)	0.06 (0.04 – 0.07)	<0.001
2 years	0.84 (0.82 – 0.85)	0.04 (0.03 – 0.05)	<0.001
3 years	0.83 (0.81 – 0.84)	0.04 (0.03 – 0.04)	<0.001
4 years	0.81 (0.80 – 0.83)	0.03 (0.02 – 0.04)	<0.001
5 years	0.81 (0.80 – 0.82)	0.03 (0.02 – 0.04)	<0.001

	Internal Testing Cohort (Manitoba) n = 23,159		
**Timeframe**	**Brier Score (95% CI)**	**Increase from Random Forest Model (95% CI)**	**p-value**
1 year	0.02 (0.02 – 0.02)	0.002 (0.002 – 0.003)	<0.001
2 years	0.04 (0.03 – 0.04)	0.003 (0.002 – 0.004)	<0.001
3 years	0.05 (0.05 – 0.05)	0.004 (0.003 – 0.004)	<0.001
4 years	0.07 (0.06 – 0.07)	0.004 (0.003 – 0.005)	<0.001
5 years	0.08 (0.08 – 0.08)	0.005 (0.003 - 0.006)	<0.001

Calibration for the Heatmap Model for Prediction of 40% Decline in eGFR or Kidney Failure at 2 years.

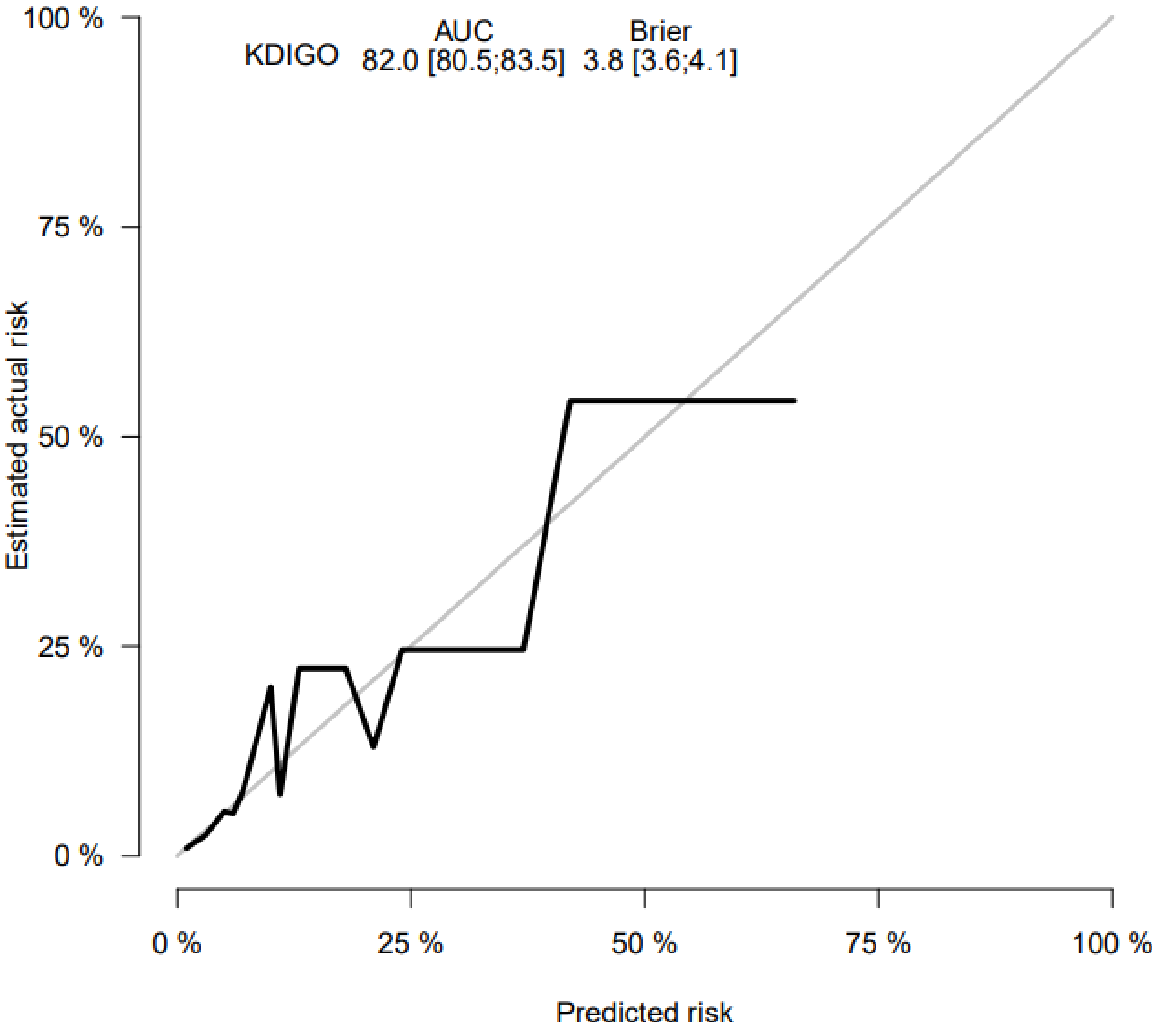

AUC and Brier Score presented as percentages.

Calibration for the Heatmap Model for Prediction of 40% Decline in eGFR or Kidney Failure at 5 years.

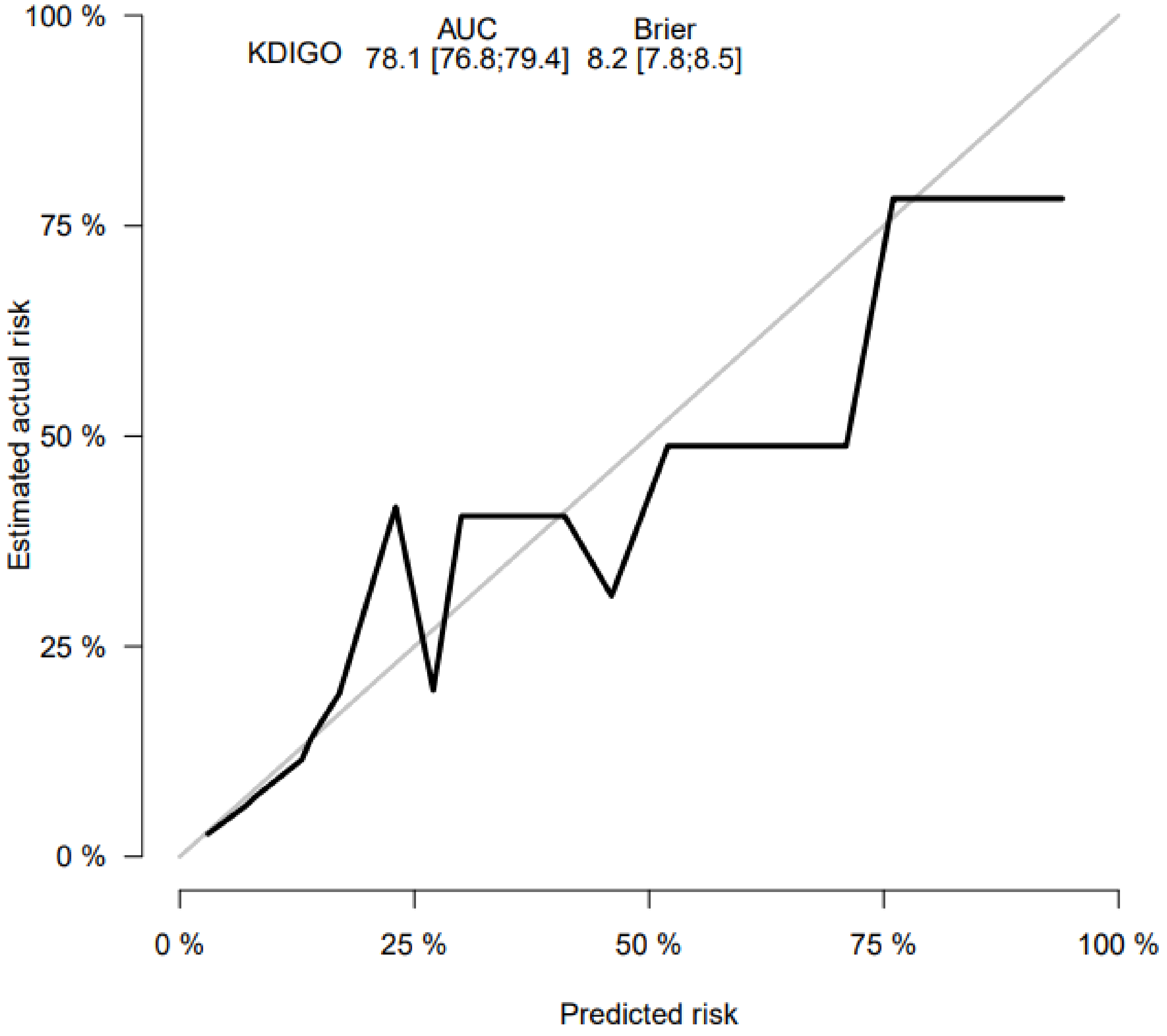

AUC and Brier Score presented as percentages.
